# Case Report: Blood Pressure Cuff–Associated Compartment Syndrome of the Right Upper Extremity in a Patient With Multiple Comorbidities

**DOI:** 10.1016/j.acepjo.2026.100344

**Published:** 2026-02-26

**Authors:** Saadman Rahman, Trey Shaughnessy, Sean McCormick

**Affiliations:** Department of Emergency Medicine, Wayne State University, Detroit, Michigan, USA

**Keywords:** blood pressure cuff, compartment syndrome, hematoma, fasciotomy, anticoagulation

## Abstract

Compartment syndrome is seen most often in patients who have experienced traumatic injuries. Rarely, compartment syndrome is caused by an atraumatic etiology. We presented a case where a 34-year-old woman developed atraumatic compartment syndrome secondary to a hematoma caused by a noninvasive blood pressure cuff inflation on the right upper extremity. Overnight, the patient experienced worsening pain and swelling, and pulses became diminished. After being evaluated by orthopedic surgery, the patient was taken to the operating room for emergent fasciotomy. A large biceps hematoma was evacuated with the return of the Doppler signal in the distal extremity. This case illustrated the importance of maintaining a high index of suspicion for potential limb ischemia and compartment syndrome even in the absence of trauma.

## 1 Background

Compartment syndrome is a condition resulting in impaired local circulation due to increased pressures leading to impaired flow out of a compartment but continued blood flow in. Without prompt treatment, increased pressure within the fascial compartment will lead to ischemia and necrosis. Compartment syndrome is most commonly associated with fractures or high-energy mechanism trauma, and rarer cases involve atraumatic presentations. Noninvasive blood pressure (NIBP) cuff–associated compartment syndrome is among these uncommon etiologies and has been reported in isolated cases, often in perioperative settings.^1^, ^2^, ^3^

We presented an interesting case of compartment syndrome of the upper extremity following an outpatient procedure in a patient with multiple comorbidities, including end-stage renal disease, antiphospholipid syndrome, complicated by prior deep venous thrombosis and pulmonary embolism, and systemic lupus erythematosus. The precipitating factor involves compression from an automated NIBP cuff, as the patient denies any other injuries or trauma. Ultimately, this case underscores the risk external compression injuries have in high-risk vascular patients and thus broadens the spectrum of settings in which NIBP cuff–associated compartment syndrome may occur.

## 2 Case

A 34-year-old woman with end-stage renal disease on hemodialysis via left upper extremity arteriovenous fistula, systemic lupus erythematosus with lupus nephritis, and antiphospholipid syndrome, complicated by prior deep venous thrombosis and pulmonary embolism on enoxaparin twice daily, presented to the emergency department with complaints of acute right upper extremity swelling and pain. Earlier that day, the patient had undergone a scheduled outpatient venoplasty of the lower extremity and had temporarily discontinued anticoagulation.

The patient reported progressive pain and swelling in her right arm soon after she had her blood pressure checked on the right upper extremity after her procedure. The patient stated that during her outpatient visit, there was repeated use of an NIBP cuff on the right upper arm. She denied any trauma, injections, or intravenous access to that extremity otherwise.

On examination of the right upper extremity, there was visible swelling and tense firmness over the biceps. The patient had preserved elbow flexion and extension on strength testing. Radial pulses were palpable, and sensation was intact. The patient’s right upper extremity did not show signs of skin discoloration. A right upper extremity venous duplex was negative for acute deep venous thrombosis, and no hematoma was noted.

Vascular surgery and orthopedic surgery were consulted, and a computed tomography angiogram was performed. Imaging demonstrated edema and enlargement of the right biceps muscle with intramuscular fluid consistent with early compartment syndrome secondary to a partial muscle tear, ischemia, contusion, or hemorrhagic blood products. Additionally, there was noted to be chronic narrowing of the right brachial artery. Vascular surgery did recommend admission for serial examinations and constant limb elevation.

The patient’s pain worsened overnight, and the radial pulse in the right upper extremity became nonpalpable and could not be obtained with bedside Doppler ultrasound. Therefore, the patient was taken to the operating room for orthopedic surgery for emergent fasciotomy. During the procedure, it was noted that there was a large intramuscular hematoma of the biceps, and it was subsequently evacuated. In the absence of any trauma, we believe the hematoma was precipitated by the repeated use of the NIBP cuff and that the hematoma was the cause of the compartment syndrome. There were preserved Doppler signals in the right hand afterwards.

The postoperative recovery for this patient was largely unremarkable, and she was ultimately discharged on hospital day 11. Since discharge, the patient has returned to the emergency department for unrelated concerns, and it was noted that her right upper extremity fasciotomy site is healing well with no ongoing neurologic deficits. The patient followed up with the orthopedic surgery clinic 1 month after the procedure and was found to have a full range of motion of her right arm with no associated paresthesias.

## 3 Discussion

NIBP cuff–associated compartment syndrome is an infrequent yet recognized complication, often observed in our anesthetized or critically ill patients. In these scenarios, prolonged NIBP cuff inflation due to labile and high systolic blood pressure,^3^ excessive cycling frequency due to tremor in the monitored extremity,^1^ or improper NIBP cuff placement across the antecubital fossa^4^ have been implicated as mechanisms leading to ischemic muscle injury. The proposed pathophysiology involves venous outflow obstruction, direct arterial compression, and increased compartment pressures caused by repetitive ischemia and reperfusion cycles. Although rare, the resulting muscle ischemia can progress rapidly to irreversible tissue damage.

This case is unique in several important respects. First, this case occurred in an outpatient rather than an intraoperative or intensive care setting, expanding the range of clinical environments in which this complication can occur. The patient’s symptoms began shortly after routine use of an NIBP cuff following a minor outpatient procedure, which underscores that even brief or seemingly benign use of automated monitoring devices can precipitate significant injury. Second, this patient’s underlying vasculopathy likely increased her susceptibility to ischemic insult. Chronic narrowing of the brachial artery, vascular changes related to end-stage renal disease, and hypercoagulability associated with antiphospholipid syndrome may have reduced tissue perfusion and impaired the ability to tolerate transient external compression. Another factor of note was the limited availability of alternative limbs for blood pressure monitoring. Because the contralateral arm contained an arteriovenous fistula, all NIBP cuff measurements were confined to the right upper extremity. Additionally, the patient’s use of anticoagulation medication increased her risk of NIBP cuff-associated hematoma. Repetitive cycling on the same limb likely compounded local ischemia, culminating in the development of compartment syndrome.

In summary, this case broadens the clinical spectrum of NIBP cuff–associated compartment syndrome to include outpatient settings and patients with preexisting vascular compromise. It also underscores the need for preventive strategies such as alternating measurement sites, minimizing NIBP cuff cycling, or using alternative monitoring methods in individuals at elevated risk. Awareness of this rare but serious complication may help clinicians intervene promptly, preventing irreversible muscle and nerve injury.

In conclusion, atraumatic upper extremity compartment syndrome can result from automated NIBP cuff utilization in patients with significant vascular comorbidities even outside of perioperative or critical care settings. This case we presented underscores the importance of a high index of suspicion, early imaging, and prompt surgical intervention to preserve patient function.

## Funding and Support

By *JACEP Open* policy, all authors are required to disclose any and all commercial, financial, and other relationships in any way related to the subject of this article as per ICMJE conflict of interest guidelines (see www.icmje.org). The authors have stated that no such relationships exist.

## Conflict of Interest

All authors declared that they have no known competing financial interests or personal relationships that could have appeared to influence the work reported in this paper.
